# COVID-19 reduced age differences in social motivation

**DOI:** 10.3389/fpsyg.2022.1075814

**Published:** 2023-01-09

**Authors:** Li Jiang, Laura L. Carstensen

**Affiliations:** ^1^Department of Marketing, George Washington University, Washington DC, United States; ^2^Department of Psychology, Stanford University, Palo Alto, CA, United States

**Keywords:** socioemotional selectivity theory, motivation, COVID-19, aging, social preferences

## Abstract

Socioemotional selectivity theory (SST) maintains that when futures loom large, as they typically do in youth, people are motivated to explore. When future time is perceived as more limited, as is typical in old age, people are motivated to pursue emotionally meaningful goals. Because the COVID-19 pandemic primed mortality across the age spectrum, it provided an opportunity to examine whether age differences in social motivation typically observed were also present during the pandemic. We measured social motivation, as operationalized by social preferences, in two studies during peak of the pandemic in 2020. Once vaccines were introduced in 2021, we conducted two additional studies using the same experimental paradigm. As hypothesized, at the peak of the pandemic, social preferences favored emotionally meaningful partners regardless of age. Social preferences differed by age (as reliably observed in research conducted before the pandemic) when vaccines were available. Findings suggest that widely documented age differences in social motivation reflect time horizons more than chronological age.

## 1. Introduction

Socioemotional selectivity theory (SST; Carstensen et al., 1999; Carstensen, 2006, 2021) was originally developed to account for “the paradox of aging,” that is, despite many hardships associated with aging, older people are in better mental health (Blazer and Hybels, 2014; Thomas et al., 2016) and report higher levels of emotional well-being than younger people (Stone et al., 2010; Carstensen et al., 2011; Burr et al., 2021). SST maintains that whereas younger people highly value exploration and novelty, with age, shrinking time horizons lead people to focus on the present, prioritize emotionally meaningful aspects of life, and invest in close social relationships. Theoretically, this shift in social motivation benefits emotional well-being.

Research examining postulates from SST has generated robust evidence for age differences in social motivation and, importantly, demonstrated that modifying future time horizons eliminates age differences (e.g., Fredrickson and Carstensen, 1990; Fung et al., 1999, 2001; Fung and Carstensen, 2006). When younger people imagine conditions that constrain future time, their social preferences mimic those observed in older people (Fredrickson and Carstensen, 1990). Conversely, when older people imagine expanded future time horizons, their preferences resemble younger adults’ (Fung et al., 1999).

In addition to hypothetical manipulations of time horizons, one report documented similar changes in younger adults’ preferences when naturally-occurring events primed life’s fragility (Fung and Carstensen, 2006) and found that younger people’s social preferences resembled those of older adults during the 2003 SARS epidemic in Hong Kong and shortly after the September 11 terrorist attacks in the United States. However, questions remain about the robustness of these effects as well as the durability of shifts as time horizons are constrained or expanded. In the present study, we examined whether age differences in social preferences were absent during COVID-19 and appeared after the COVID-19 vaccines were available, as COVID-19 imposed ubiquitous threats to life at its peak which receded when vaccines became available.

To assess social motivation, we surveyed younger, middle-aged, and older Americans twice during the peak of the COVID-19 pandemic and two more times after vaccines had become available. The survey was based on the measure of social preferences used in previous research Fredrickson and Carstensen (1990) and Fung and Carstensen (2006). We reasoned that well-documented age differences in social preferences would be absent during the pandemic but would be evident after vaccines were introduced.

The COVID-19 pandemic first surged in the United States during April and May of 2020. Hundreds of thousands of Americans contracted the virus during this window of time. The life-threatening nature of the pandemic primed mortality across the age spectrum. Many young people reported feeling older when the pandemic first surged (Heller, 2020). According to SST, such conditions are associated with preferences for emotionally meaningful goals. Thus, in April 2020 and subsequently May 2020, we deployed a survey to Americans aged 18–93. We hypothesized that younger, middle-aged, and older adults would express similar preferences as older adults during the peak of the pandemic.

In December 2020, the first dose of an FDA-authorized COVID-19 vaccine was delivered to all 50 states (Cullinane et al., 2020) and vaccination programs were put in place across the nation. People began to see a return to normalcy. We reasoned that at this point, younger adults would again begin to perceive their futures as expansive. After vaccination efforts were underway, we deployed the survey in two samples, once in March 2021 and again in June 2021. Thus, we tested and replicated the hypotheses in a total of four independent samples; two samples during the peak of the pandemic and two more once vaccines had become available.

## 2. Materials and methods

### 2.1. Participants

The study was approved by Institutional Review Board at George Washington University. We preregistered the hypotheses and measures before collecting any data. As noted in our preregistration,^1^ we planned to collect data during the peak and after the COVID-19 outbreak is under control. We recruited online cross-sectional samples of adults who were currently living in the United States, who had not contracted COVID-19 and whose family had not been infected with COVID-19. Informed consent was obtained from all participants. We performed a power analysis based on the effect sizes from previous studies (e.g., Fung and Carstensen, 2006). To detect an effect size of risk difference = 0.25 (based on past studies the proportion of participants who chose the emotionally closer partner largely ranged from 50% to 75% so the risk difference was 25%), 58 participants per condition would be required to have 80% power and roughly 100 participants per condition would be required to have 95% power. Sample 1 was recruited during the first peak of COVID-19 from the Cloud Research platform (also called TurkPrime)^2^ and an online subject pool at a northeastern university in the United States at the same time (*N* = 254). Because participants’ average age on the Cloud Research platform was 35 years old and the sample was screwed toward middle-aged and older adults, the university sample was recruited at the same time to complement the Cloud Research sample to balance the number of participants in each age group. From the Cloud Research sample in Sample 1, we learned the distribution of younger, middle-aged, and older adults on the platform, and in subsequent studies, we decided to just increase the total sample size to meet our research goal of collecting at least 100 participants per age group. Sample 2 (*N* = 577) served as a replication of Sample 1, and was recruited *via* the Cloud Research during the peak of COVID-19. Sample 3 (*N* = 527) was recruited when the vaccines were available and *via* the Cloud Research platform. Sample 4 served as a replication of Sample 3 and was recruited *via* Prolific (*N* = 490).^3^ In all the samples, participants completed a study titled “Attitude towards the COVID-19 pandemic” as part of a large survey package and that was always presented as the first study in the survey package. Participants from the northeastern university received 0.50 course credit, and participants from the Cloud Research and the Prolific platforms received $1 for compensation. Data were collected using the Qualtrics survey software during the peak of the pandemic, April 2020 (the initial survey) and May 2020 (the replication), and after the COVID-19 vaccines were available, March 2021 (the initial survey) and June 2021 (the replication). We parsed the continuous age into three age groups: younger adults (18–35 years old), middle-aged adults (36–59 years old), and older adults (60 years old and above), and preregistered a plan to collect 100 participants for each age group.^4^ We hypothesized that during the initial outbreak of COVID-19, younger, middle-aged, and older adults would adopt emotionally meaningful goals that would be reflected in preferences for emotionally close social partners. We hypothesized that age differences in social preferences would be observed once the vaccines had become available. We did not analyze data until data collection was completed. For each sample, all participants were included in the data analyses. We report all the preregistered manipulations and measures. For brevity, some results are reported in the Supplementary materials.

### 2.2. Procedure and measures

Surveys were conducted twice to provide replications at each phase of the pandemic. One survey and one replication study were administered in spring 2020 at the first peak of the COVID-19 pandemic. One survey and one replication study were then administered in the spring of 2021 after COVID-19 vaccines were available. Only participants who had not contracted COVID-19 and whose family had not been infected were recruited for the surveys. The focal measure (presented below) was included as part of a larger survey that assessed people’s attitude toward COVID-19. The surveys resulted in another working paper (Jiang et al., 2021).

Participants first read a description of the COVID-19 pandemic (the same description was presented for all four samples): “Coronaviruses are a large family of viruses that are common in people and many different species of animals, including cats, and bats. The complete clinical picture with regard to COVID-19 is not fully known. Reported illnesses have ranged from very mild (including some with no reported symptoms) to severe, including illness resulting in death.” Then, participants completed the primary measure of social partner preferences in which relative preferences for emotionally meaningful over information-focused goals were assessed (Fredrickson and Carstensen, 1990). Specifically, participants were presented with the following question: “Imagine that you have half an hour of free time, with no pressing commitment. You have decided that you would like to spend this time with another person. Assuming that the following three people are available to you for an online chat, which person would you choose to spend that time with?” Participants were presented with three prospective social partner options and asked to choose one of them. One of the options was an emotionally close partner, “a member of your immediate family you have not recently seen.” The other two were focused on exploration: “a recent acquaintance with whom you seem to have much in common” and “an author of a book you have read.”

In the larger survey package, participants also answered one question about the extent to which they had questioned the meaning of life in the past month. The question is not relevant to the focal study and we presented the measures and results in the Supplementary materials.^5^ Finally, participants answered questions about their age, gender, education, income, ethnicity, and number of people in their household.

On the second survey of 2021 when vaccines were made widely available, participants also self-reported whether they had been fully vaccinated (1 = *yes, fully vaccinated*; 2 = *I have taken only one dose of vaccine and have to take another dose*; 3 = *No, but I will take the vaccine*; 4 = *No, but I will not take the vaccine*). We recoded participants choosing “2” to “4” as “0” to indicate they were not fully vaccinated, so the variable *vaccinated* was a binary variable with 1 as *“fully vaccinated”* and 0 as *“not fully vaccinated*.*”*^6^

## 3. Results

All analyses were conducted using SAS 9.4.

### 3.1. Preliminary analyses

Table 1 includes sample characteristics. Across the four samples, participants ranged in age from 18 to 93 years (*M* = 45.15, *SD* = 16.79). We classified participants into three age groups: younger adults (18–35 years old), middle-aged adults (36–59 years old), and older adults (60 years old and above). 76 to 80% of participants identified as White. The median household income for all four samples was between $50,000 and $59,999, which is comparable with the median U.S. income of $65,712 (U.S. Census Bureau, 2019a). Across the four samples, 20.1 to 22.5% reported living alone, which is comparable with U.S. Census’ statistics that 28% of American households had one occupant (U.S. Census Bureau, 2019b). Table 2 presents Pearson’s correlations between age and other demographic variables in four samples.

**Table 1:** Age, gender, ethnicity, household structure, and education by subsample.

	First peak of COVID-19		After COVID-19 vaccines were available	
	Sample 1	Sample 2	Sample 3	Sample 4
	Initial Survey 2020	Replication Survey 2020	Initial Survey 2021	Replication Survey 2021
Age (%)				
18–35	39.8	28.2	26.8	42.7
36–59	25.6	44.9	43.9	30.2
60 or above	34.6	26.9	29.3	27.0
Gender (% Female)	56.7	51.9	53.7	52.9
White (%)	76.3	80.0	79.4	77.3
Live alone (%)	21.74	21.1	22.5	20.1
At least two-year college (%)	60.1	73.6	73.9	67.9

**Table 2:** Pearson’s correlations between age and other variables in four samples.

	Sample 1 Initial survey at peak of pandemic	Sample 2 Replication survey at peak of pandemic	Sample 3 Initial survey when vaccines were available	Sample 4 Replication survey when vaccines were available
	Age	Age	Age	Age
Race (White)	0.34	0.25	0.18	0.18
	*p <* 0.0001	*p <* 0.0001	*p <* 0.0001	*p <* 0.0001
Employed (Yes)	−0.03	−0.34	−0.41	−0.24
	*p =* 0.61	*p <* 0.0001	*p <* 0.0001	*p <* 0.0001
Education	0.28	−0.03	−0.03	0.09
	*p <* 0.0001	*p =* 0.47	*p =* 0.46	*p =* 0.05
Live alone (Yes)	0.27	0.08	0.06	0.25
	*p <* 0.0001	*p* = 0.07	*p* = 0.16	*p <* 0.0001
Gender (Male)	−0.06	0.12	0.13	0.13
	*p* = 0.38	*p* = 0.004	*p* = 0.003	*p* = 0.003

Sample 1 included undergraduate students from a university so the demographics are a bit different from the participants on Cloud Research or Prolific.

### 3.2. Primary analyses

#### 3.2.1. Age group and social preferences

**Figure 1:**
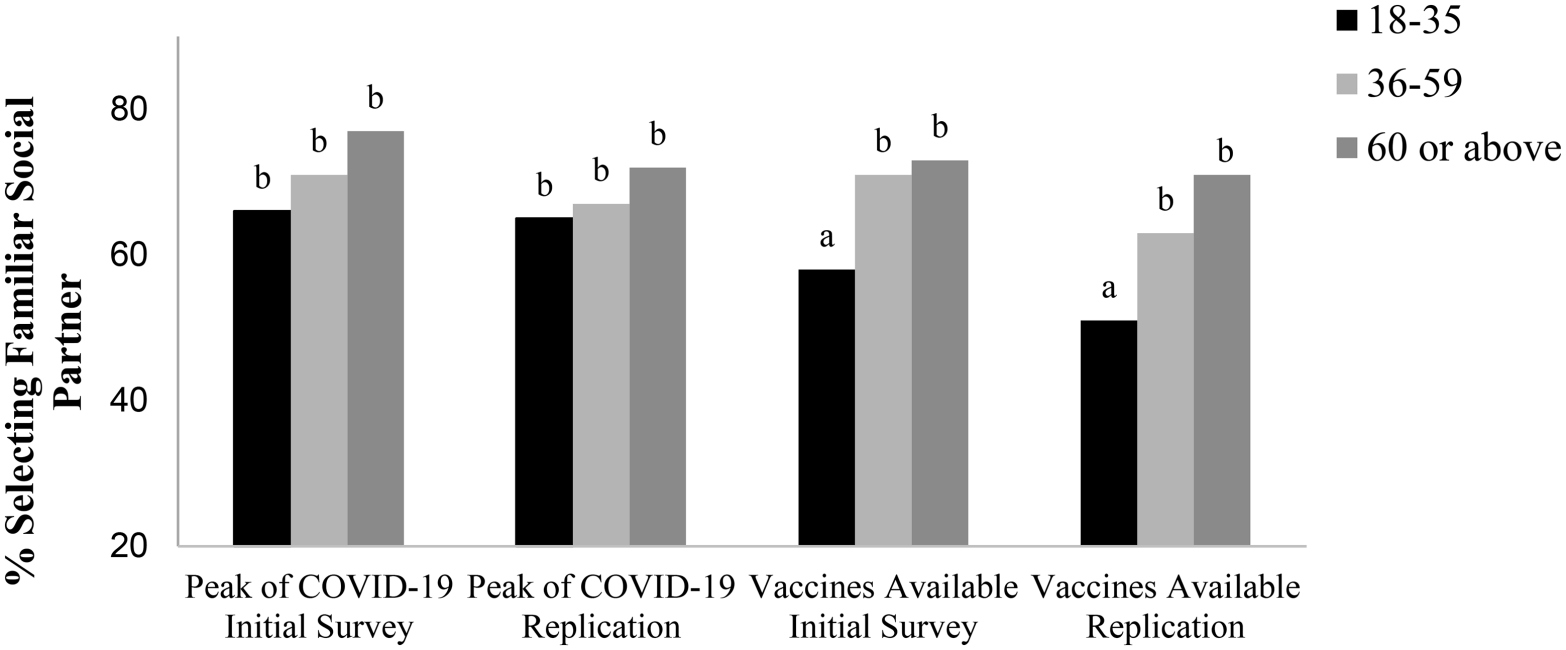
Percent selecting the emotionally close partner by age groups (18–35, 36–59, 60 or above years old) during the peak of the pandemic (two replicated studies) and after the vaccines were made available (two replicated studies). For each sample, percentages with different subscripts were significantly different at *p* < 0.05.

We examined social preferences by age groups during COVID-19 and after the vaccines were available. Logistic regressions were conducted on social preferences with age groups as categorical variables. We used two dummy variables, *Young* and *Middle* to code the three age groups. Older adults were treated as the reference group and were coded as *Young = 0 and Middle = 0*. Middle-aged adults were coded as *Young = 0 and Middle = 1*. Younger adults were coded as *Young = 1 and Middle = 0*. The dependent variable was recoded as a binary variable: 1 for emotionally close partner, 0 for the other two options on distant partners. Figure 1 shows the percentages of participants who chose the emotionally close social partner by age groups during the first peak of COVID-19 or after the vaccines were available. Previous literature has consistently shown that younger adults’ preferences are typically distributed evenly across the social partner options and younger adults are less likely to choose the emotionally close social partners compared to middle-aged and older adults (e.g., Fredrickson and Carstensen, 1990; Carstensen and Fredrickson, 1998; Fung and Carstensen, 2006). However, on both surveys administered during the peak of the pandemic, younger adults preferred emotionally close social partners as much as middle-aged and older adults. Specifically, on the first survey, a logistic regression revealed no significant difference between the three age groups on social preferences, Wald *χ*^2^(2, *N* = 254) = 2.58, *p* = 0.276. 66% of younger adults, 71% of middle–aged adults, and 77% of older adults preferred to interact with a familiar social partner. Similarly, on the replication survey, there was no significant difference between three age groups on social preferences, Wald *χ*^2^(2, *N* = 577) = 1.644, *p* = 0.440. 65% of younger adults, 67% of middle-aged adults, and 72% of older adults chose to interact with a familiar social partner.

In contrast, logistic regressions revealed that the expected age differences in social preferences were observed 4 months [Wald *χ*^2^(2, *N* = 527) = 8.84, *p* = 0.012] and the results were replicated 6 months [Wald *χ*^2^(2, *N* = 490) = 13.35, *p* = 0.001] after the introduction of vaccines in the United States. Pairwise comparisons showed that in March 2021, 4 months after the vaccines were available, younger adults (58%) were less likely to choose the emotionally close social partner than older adults (73%), Wald *χ*^2^(1, *N* = 295) = 7.06, *p* = 0.008, odds ratio = 0.517, 95% CI = [0.316, 0.839], and middle-aged adults (71%), Wald *χ*^2^(1, *N* = 372) = 6.39, *p* = 0.012, odds ratio = 0.568, 95% CI = [0.366, 0.881]. Middle-aged adults were as likely to select the emotionally close social partner (71%) as were older adults (73%), Wald *χ*^2^(1, *N* = 386) = 0.166, *p* = 0.683, odds ratio = 0.910, 95% CI = [0.575, 1.429]. In the replication survey using Sample 4, younger adults (51%) were less likely to choose the emotionally close social partner than older adults (71%), Wald *χ*^2^(1, *N* = 342) = 12.48, *p* = 0.0004, odds ratio = 0.435, 95% CI = [0.272, 0.687], and middle-aged adults (63%), Wald *χ*^2^(1, *N* = 357) = 4.74, *p* = 0.030, odds ratio = 0.620, 95% CI = [0.402, 0.951]. Middle-aged adults (63%) were as likely to select the emotionally close social partner as were older adults (71%), Wald *χ*^2^(1, *N* = 281) = 1.92, *p* = 0.165, odds ratio = 0.702, 95% CI = [0.424, 1.155]. Among people who had been fully vaccinated (*N* = 348), this age difference persisted (Younger: 49%, Middle-aged: 65%, Older: 73%, Wald *χ*^2^(2, *N* = 348) = 15.16, *p* = 0.0005). However, among people who had not been fully vaccinated (*N* = 142), there was no difference in social preferences in all three age groups (Younger: 57%, Middle-aged: 57%, Older: 65%, Wald *χ*^2^(2, *N* = 142) = 0.676, *p* = 0.713). Pairwise comparison demonstrated that among fully vaccinated people, younger adults (*N* = 151) were less likely to choose the emotionally close social partner than older adults (*N* = 96), 49% vs.73%, Wald *χ*^2^(1, *N* = 247) = 13.39, *p* = 0.0003, odds ratio = 0.357, 95% CI = [0.203, 0.614], and middle-aged adults (*N* = 101), 49% vs. 65%, Wald *χ*^2^(1, N = 262) = 6.47, *p* = 0.011, odds ratio = 0.510, 95% CI = [0.303, 0.857]. Middle-aged adults were as likely to select the emotionally close social partner as were older adults, 65% vs. 73%, Wald *χ*^2^(1, N = 197) = 1.314, *p* = 0.252, odds ratio = 0.700, 95% CI = [0.379, 1.284]. However, these effects did not occur for people who had not been fully vaccinated. These results suggest that vaccines returned the age differences in social preferences.

#### 3.2.2. Treating age as a continuous variable

To examine our hypothesis, we first examined the correlations of age and other variables in each sample (see Table 2). Race, employment status, education, gender, and whether the participant was living alone were correlated with age, so we included these variables in the logistic regression models below.

For each sample, we ran a series of binary logistic regressions, regressing the recoded social preferences (1 = familiar social partner; 0 = novel social partner) on age as a continuous variable (see Table 3). For each sample, in the first model, we examined age effects on choices of emotionally close others. We did not find age differences on preferences for emotionally close others in the initial survey or the replication when the pandemic was peaking in the United States [Table 3 columns (1) and (3)]. However, we observed significant age differences for preferences for emotionally close others in the two surveys conducted when vaccines had become available [Table 3 columns (5) and (7)]. In the second model, we then added demographic variables that were significantly correlated with age (i.e., race, living alone, education, gender, and employment status). Findings were unchanged for surveys conducted during the pandemic peak and the vaccination phase of the pandemic [Table 3 columns (2), (4), (6), and (8)].

**Table 3:** Logistic regressions: age effects on preferences of emotionally close others.

	Peak of COVID-19				After vaccines were available			
Variable	Sample 1 Initial survey (*N* = 254)		Sample 2 Replication (*N* = 577)		Sample 3 Initial survey (*N* = 527)		Sample 4 Replication (*N* = 490)	
	(1)	(2)	(3)	(4)	(5)	(6)	(7)	(8)
	Coefficient	Coefficient	Coefficient	Coefficient	Coefficient	Coefficient	Coefficient	Coefficient
Intercept	0.370	−0.261	0.303	0.715	−0.055	−0.320	−0.580*	−1.547***
Age	0.012	0.015	0.010	0.011	0.017**	0.020**	0.024***	0.022***
Race (White)		−0.135		−0.006		−0.265		0.089
Employed (Yes)		0.230		−0.041		−0.453		0.204
Education		0.128		−0.100		0.015		0.123
Live alone (No)		−0.833*		−0.646**		−0.071		−0.373
Gender (Male)		0.209		0.293		0.615**		0.947***

The dependent variable in the binary logistic regression is the log odds of the choice of the emotionally close partner. Terms in parentheses after variable names denote the reference level. The dependent (predicted) variable and the binary predictors (race, employed, live alone, and gender) were unstandardized.

* *p* < 0.05;

** *p* < 0.01;

*** *p* < 0.001.

## 4. Discussion

Findings suggest that during the peak of the COVID-19 pandemic when mortality was highly salient in the U.S. population, the social preferences of younger adults were indistinguishable from preferences of middle-aged and older adults. Regardless of age, people expressed preferences for emotionally close social partners over novel social partners. After vaccines had become available, younger people’s preferences returned to well-documented pre-pandemic preferences for interactions with novel social partners who satisfied exploratory goals. Because surveys were deployed and replicated during each phase of the pandemic, findings appear to be robust and reliable.

Because the pandemic primed endings for a prolonged period, it allowed us to address an important theoretical issue about emotional aging, namely, does a mortality threat change younger adults’ goals in ways that resemble those expressed by older adults? And if so, do such changes endure? Our findings suggest that the answer to the first question is yes and the answer to the second is no. After vaccines had become available, well-documented age differences in social goals reappeared. Presumably, vaccines allowed younger people to focus once again on their long-term futures.

Theoretically, the fluidity of preferences underscores the importance of time horizons in social motivation. Age differences characterized by smaller social networks comprised of emotionally close social partners—long thought to reflect cognitive and physiological decline when observed in older people—are likely related to motivational differences associated with constraints on future time. The fact that vaccine availability in the second pair of surveys found that younger people were placing priority on future-oriented goals offers strong support for the role of perceived time horizons on social preferences.

In addition to theoretical implications, findings hold practical importance. Social goals change in the face of life-threatening disease. Understanding changes to social priorities can enable policymakers and health practitioners to tailor public health messages aimed at slowing the spread of infectious diseases to match goals of target audiences (Carstensen and Hershfield, 2021). Whereas surface-level market segmentation tends to rely on time since birth (i.e., chronological age), perceived time left in life may be more informative in explaining the systematic age differences in motivation, especially in times of mortality threat. Indeed, the pandemic led younger people to feel older than their chronological age (Heller, 2020) and instigated smaller emotionally dense social networks (Williams, 2021).

One limitation of the study is the absence of a pre-pandemic baseline. However, the patterns are strikingly similar to past studies based on the same experimental paradigm (e.g., Fung and Carstensen, 2006). Further, by surveying two independent samples during each phase of the pandemic, the findings underscore the robustness of the pattern. Our plan is to recruit another sample after the pandemic fully ends and compare those results with findings from the present study. Another limitation is that we did not directly assess future time perspective (FTP). One alternative explanation is that younger adults chose their close family member not because of perceived mortality threat to themselves but as a result of the threat to their older relatives (e.g., parents or grandparents). In Sample 2 (but not in every sample), after participants indicated their choice of close or distant partners, we collected exploratory open-ended responses on why participants made their choices. To address the above alternative explanation, we further examined the open-ended responses. None of the participants mentioned the mortality threat to their older relatives. Instead, most participants mentioned that family made them feel calm and protected. Most participants only mentioned their family in general. However, among participants who mentioned specific family members, younger adults were most likely to mention their wife/husband, followed by sister/brother/cousin, followed by parents, grandparents, and children. Thus, these results provide preliminary evidence that it is less likely that younger adults chose their close family member as a result of perceived mortality threat to their older relatives. Rather, they were thinking about threat to their mortality. We encourage future research to directly measure future time perspective.

In summary, these findings are among the first to examine social preferences across a prolonged exposure to life-threatening conditions. Future research may examine whether there is a duration threshold for the prolonged exposure and the threshold value. We also demonstrate the anticipation that life-threatening conditions will end restores younger adults’ motivational preferences. Findings add to evidence that age differences in social preferences are adaptive and shaped by reminders of the fragility of life.

## Data availability statement

The original contributions presented in the study are included in the article/Supplementary material, further inquiries can be directed to the corresponding author.

## Ethics statement

The studies involving human participants were reviewed and approved by George Washington University Institutional Review Board. The patients/participants provided their written informed consent to participate in this study.

## Author contributions

LJ developed the study concept, designed the study, and collected and analyzed the data and drafted the manuscript. LC revised the manuscript. All authors contributed to the article and approved the submitted version.

## Funding

Partial support for the project was provided by NIA grant R37-8816 to LC.

## Conflict of interest

The authors declare that the research was conducted in the absence of any commercial or financial relationships that could be construed as a potential conflict of interest.

## Publisher’s note

All claims expressed in this article are solely those of the authors and do not necessarily represent those of their affiliated organizations, or those of the publisher, the editors and the reviewers. Any product that may be evaluated in this article, or claim that may be made by its manufacturer, is not guaranteed or endorsed by the publisher.

## Footnotes

^1^ https://aspredicted.org/PLX_IFJ

^2^ https://www.cloudresearch.com

^3^We used Prolific to collect data in June 2021 because the Cloud Research got incredibly slow at that time to collect data.

^4^Due to distribution of age groups in specific subsamples, the specific numbers of participants collected for each sample varied somewhat. Except for the first sample, all other samples had more than 100 participants in each age group.

^5^Because the primary measures were incorporated into a large survey package, inclusion of the whole survey package in the method section is not always possible. We presented the primary measures in the manuscript and the additional measures in the Supplementary materials.

^6^We did not measure whether each participant was vaccinated in the initial survey deployed in March 2021 (Sample 3), because at that time the vaccines were only offered to high-risk people (e.g., older adults and medical providers) and the vaccination rate in the population had only reached 23%.
