# Supplementary material for: COVID-19 reduced age differences in social motivation
Source: Front Psychol. 2023 Jan 9;13:1075814. doi: 10.3389/fpsyg.2022.1075814 (PMC9870591; doi:10.3389/fpsyg.2022.1075814)
Supplement: Supplementary file 1 [file Presentation_1.pdf]

# COVID-19 Reduced Age Differences in Social Motivation

## Online Supplemental Materials

In the supplemental material, we provided additional measures and results.

## METHOD

We measured the extent to which participants thought about the meaning of life in the four samples: "How often, if at all, have you thought about the meaning and purpose of life in the past week?", 1 = *often*; 4 = *never*). The question was taken from the World Values Survey (Inglehart et al., 2018).

## RESULT

*Thinking about the meaning and purpose of life.* We reverse-coded the scale so higher numbers indicate greater tendencies to question the meaning and purpose of life. In the first pandemic phase survey, there was not a significant difference by age in the tendency to think about meaning and purpose of life ($F(2, 251) = 1.29$, $p = .278$) or the pandemic phase replication survey in 2020 ($F(2, 574) = 1.29$, $p = .276$); nor were age differences observed during the first survey deployed during the vaccination phase ($F(2, 524) = 0.10$, $p = .90$), at the point when 23% of the population had been vaccinated. Age differences were observed in the fourth survey ($M_{young} = 3.14$, $M_{middle\text{-}aged} = 3.00$, $M_{old} = 2.89$, $F(2, 487) = 4.25$, $p = .015$) after vaccines had become available to all.  In the fourth survey, younger adults ($M_{young} = 3.14$) were more likely to question the meaning of life than older adults ($M_{old} = 2.89$, $t(340) = 2.86$, $p = .004$). There were no differences between middle-aged and older adults ($t(279) = 1.17$, $p = .24$), or between younger and middle-age adults ($t(355) = 1.66$, $p = .098$). As previous research has demonstrated, younger adults typically question the meaning of life more than older adults (Alter and

Hershfield, 2014); these results suggest that as vaccines became widely available, younger adults' preferences gradually resumed to the pre-pandemic level.

**Additional References**

Alter, A. L., and Hershfield, H. E. (2014). People search for meaning when they approach a new decade in chronological age. Proceedings of the National Academy of Sciences, 111(48), 17066-17070.

Inglehart, R., C. Haerpfer, A. Moreno, C. Welzel, K. Kizilova, J. Diez-Medrano, M. Lagos, P. Norris, E. Ponarin & B. Puranen et al. (eds.). 2018. World Values Survey: Round Six - Country-Pooled Datafile. Madrid, Spain & Vienna, Austria: JD Systems Institute & WVSA Secretariat. doi.org/10.14281/18241.8
